# Brevilin A, a Sesquiterpene Lactone, Inhibits the Replication of Influenza A Virus In Vitro and In Vivo

**DOI:** 10.3390/v11090835

**Published:** 2019-09-08

**Authors:** Xiaoli Zhang, Yiping Xia, Li Yang, Jun He, Yaolan Li, Chuan Xia

**Affiliations:** 1 Department of Biotechnology, College of Life Science and Technology, Jinan University, Guangzhou 510632, Guangdong, China; 2 Institute of Laboratory Animal Science, Jinan University, Guangzhou 510632, Guangdong, China; 3 Institute of Traditional Chinese Medicine and Natural Products, Jinan University, Guangzhou 510632, Guangdong, China

**Keywords:** influenza A virus, brevilin A, antiviral, sesquiterpene lactone, replication

## Abstract

With the emergence of drug-resistant strains of influenza A viruses (IAV), new antivirals are needed to supplement the existing counter measures against IAV infection. We have previously shown that brevilin A, a sesquiterpene lactone isolated from *C. minima*, suppresses the infection of influenza A/PR/8/34 (H1N1) in vitro. Here, we further investigate the antiviral activity and mode of action of brevilin A against different IAV subtypes. Brevilin A inhibited the replication of influenza A H1N1, H3N2, and H9N2 viruses in vitro. The suppression effect of brevilin A was observed as early as 4–8 hours post infection (hpi). Furthermore, we determined that brevilin A inhibited viral replication in three aspects, including viral RNA (vRNA) synthesis, expression of viral mRNA, and protein encoded from the M and NS segments, and nuclear export of viral ribonucleoproteins (vRNPs). The anti-IAV activity of brevilin A was further confirmed in mice. A delayed time-to-death with 50% surviving up to 14 days post infection was obtained with brevilin A (at a dose of 25 mg/kg) treated animals compared to the control cohorts. Together, these results are encouraging for the exploration of sesquiterpene lactones with similar structure to brevilin A as potential anti-influenza therapies.

## 1. Introduction

Influenza A viruses (IAV) are a major cause of respiratory infection in humans and are responsible for significant morbidity and mortality worldwide. Annually, seasonal influenza epidemics affect approximately 5%–15% of the global population, resulting in 290,000–650,000 deaths [1,2]. Vaccination programs are important for preventing and controlling influenza. However, the efficacy of vaccination is typically only 40%–60% and can be lower than 20% during years of vaccine mismatch [3]. Thus, antiviral therapies are a vital option for the treatment of influenza.

Until now, three types of antivirals have been approved by the FDA for influenza prevention and therapies, including M2 ion channel inhibitors (i.e., adamantanes and rimantadine) [4], neuraminidase inhibitors (NAIs, i.e., oseltamivir and zanamivir) [5], and a cap-dependent endonuclease inhibitor [6,7]. However, the M2 inhibitors are no longer used clinically as currently almost all circulating IAV strains are resistant to adamantanes [8]. Moreover, the 2008–2009 seasonal H1N1 influenza virus strain in North America presented nearly complete resistance to oseltamivir [9]. Baloxavir marboxil was approved for treating influenza last year, but recent work has shown that viral resistance is still a concern [6,10,11]. Amidst concerns about drug resistance, the development of novel antivirals with distinct mechanisms of action is necessary.

**Figure 1:**
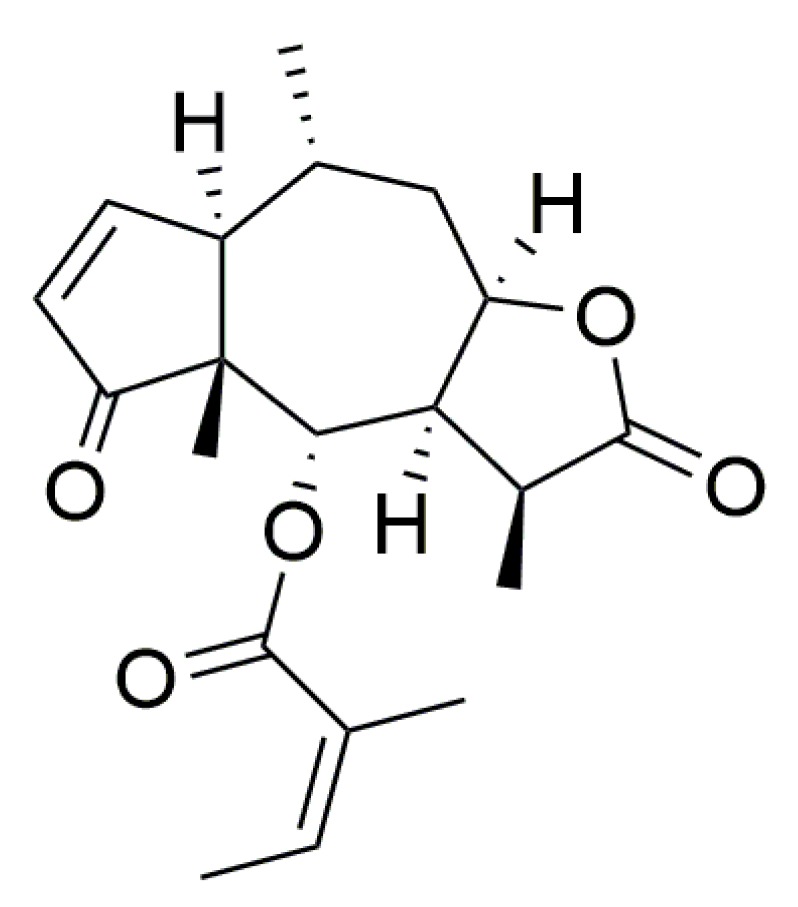
The chemical structure of brevilin A.

Brevilin A (chemical structure shown in Figure 1) is a sesquiterpene lactone isolated from medicinal herb *Centipeda minima*. As a major constituent of *C. minima* [12], it has been reported that brevilin A displays multiple activities such as anti-tumor [13,14,15,16,17], anti-bacterial [18], and antiprotozoal [19]. We previously evaluated the antiviral activity of 16 sesquiterpene lactones isolated from *C. minima* against influenza A/PR/8/34 (H1N1) virus in vitro. Eight of them showed significant antiviral activity. Among them, brevilin A exhibited the strongest antiviral effect [20], but the mechanism of this antiviral effect was not extensively studied. Here, we extend our previous findings by investigating the antiviral effects of brevilin A against various IAVs and mode of actions in vitro at a noncytotoxic concentration. We found that brevilin A exhibits significant antiviral activities against all tested IAV strains, and it inhibits the vRNA synthesis and the expression of some viral proteins. Furthermore, the anti-IAV effect of this compound in vivo was also evaluated.

## 2. Materials and Methods

### 2.1. Compounds and Reagents

Brevilin A (purity >95% by HPLC) was isolated from the supercritical fluid extract of *C. minima*. Ribavirin was purchased from Sigma-Aldrich (St. Louis, MO, USA). Both compounds were dissolved in DMSO to prepare a solution with the concentration of 50 mM and stored at −20 °C for in vitro experiments. Brevilin A did not show cytotoxicity in Madin–Darby canine kidney (MDCK) epithelial cells up to 8 µM, which was used as the maximum concentration for in vitro antiviral assays. For in vivo experiments, brevilin A was dissolved in 10% Lipovenos containing 0.2% DMSO, 10% PEG300, and 2.5% glycerol, while oseltamivir carboxylate (Tamiflu, Roche, Basel, Switzerland), purchased from Guangzhou Overseas Chinese Hospital (Guangzhou, China), was dissolved in distilled water. Leptomycin B (LMB, a nuclear export inhibitor) solution was obtained from Beyotime Institute of Biotechnology, Shanghai, China.

Mouse anti-IAV NP (ab128193) and M2 (ab5416) antibodies, mouse anti-GAPDH antibody (ab181603), and donkey anti-mouse lgG (H + L) secondary antibody (ab150105) were purchased from Abcam Company Ltd., Shanghai, China. Mouse anti-IAV HA (GTX28262), NA (GTX629696) and M1 (GTX125928) antibodies, rabbit anti-IAV NS1 (GTX125990) and NS2 (GTX125953) antibodies were obtained from GeneTex, Alton Pkwy Irvine, CA, USA.

### 2.2. Cells and Viruses

Madin–Darby canine kidney (MDCK) epithelial cells (obtained from the Key Laboratory of Veterinary Vaccine Innovation of the Ministry of Agriculture, P. R. China) were maintained as monolayers in Dulbecco’s modified Eagle’s medium (DMEM, Gibco, Beijing, China) supplemented with 10% fetal bovine serum (FBS, Gemini, Calabasas, CA, USA), and 1% penicillin/streptomycin (Pen/Strep, Gibco) at 37 °C, 5% CO_2_.

Influenza A/PR/8/34 H1N1 (PR8, a gift from the Key Laboratory of Veterinary Vaccine Innovation of the Ministry of Agriculture, P. R. China), A/FM/1/47 H1N1, A/Hong Kong/498/97 H3N2, and A/chicken/Guangdong/1996 H9N2 viruses were grown in 10-day-old chicken embryos, titrated on MDCK cells, and then stored at −80 °C. For virus infection, cells were washed with PBS and infected with virus in PBS containing 0.3% BSA (Sigma) and 1% Pen/Strep for 1 h at 37 °C. The inoculum was aspirated, and cells were incubated in infection medium supplemented with DMEM, 0.3% BSA, 2 μg/mL TPCK-trypsin (Sigma-Aldrich), and 1% Pen/Strep.

### 2.3. Animals

Female BALB/c mice, six to eight-week-old (average weight, 16.0 ± 2.0 g), were obtained from Beijing Vital River Laboratory Animal Technology Co., Ltd. (Beijing, China). The mice were quarantined 2–3 days prior to the experimental manipulation and were fed standard rodent chow and had ad libitum access to water. Animal experiments were conducted under the guidance of both the Guangdong Provincial Center for Disease Control and Prevention’s Institutional Animal Care and Use Committee. The protocols were approved by South China Agricultural University’ committee on the Ethics of Animal Experiments of Animal Biosafety Level 3 (permit no. 2017A002).

### 2.4. Plaque Assay and Plaque Reduction Assay

Plaque assay was performed as described previously [21]. Briefly, MDCK cells (8 × 10^5^/well) were seeded into 6-well plates and incubated overnight. The infected cells were overlaid with F12-DMEM (Gibco) containing 2% Oxoid agar, 0.2% BSA, DEAE dextran, and 2 µg/mL TPCK-treated trypsin, and further incubated for 48–72 h. The cells were fixed with 4% paraformaldehyde for 30 min and then stained with 0.1% crystal violet. Virus titers were determined by counting the PFU (plaques) for each sample and expressed as PFU/mL.

The plaque reduction assay was performed to determine EC_50_, as described previously [22]. Briefly, monolayer MDCK cells were incubated with virus (~50 pfu/well) at 37 °C for 1 h, rocking every 15 min. Cells were washed twice with PBS and an agar overlay with or without brevilin A (0.5–8 µM) or ribavirin (5–30 µM) was added to each well. After 48–72 h incubation, the cells were fixed and plaques were counted by crystal violet staining. The concentration required to reduce the plaque number by 50% (EC_50_) was calculated using the log (inhibitor) versus response logistic nonlinear regression equation in Graphpad Prime 6.0 software (LaJolla, CA, USA).

### 2.5. Immunofluorescence

At the indicated time points after infection, MDCK cells were washed with PBS three times and fixed in 4% paraformaldehyde for 15 min at 4 °C and permeabilized with 0.25% triton in PBS for 15 min at room temperature, and then incubated for 1 h with anti-NP (1:100) monoclonal antibody. After washing with PBS, the cells were incubated for 1 h with the secondary antibodies, Alexa Fluor 488-conjugated goat anti-mouse lgG (H + L) (1:1000; Thermo Fisher, Waltham, MA, USA) antibody. Nuclei were counterstained with DAPI. Cells were observed under the Leica confocal microscope.

### 2.6. Western Blot Assay

Confluent MDCK cell monolayers were first infected with PR8 (MOI = 1), and then treated with brevilin A or vehicle. At the indicated time of 4 or 8 hour-post-infection (hpi), proteins from total cell lysates were separated using SDS-PAGE and transferred onto a polyvinylidene difluoride membrane (Millipore, Burlington, MA, USA). The membrane was blocked with 5% nonfat milk or 5% BSA in PBS containing 0.1% Tween 20 and incubated overnight at 4 °C with primary mouse anti-HA (1:1000), mouse anti-NA (1:1000), mouse anti-M1 (1:3000), mouse anti-M2 (1:1000), rabbit anti-NS1 (1:1000), rabbit anti-NS2 (1:1000), and mouse anti-NP (1:1000) antibody, or mouse anti-GAPDH and mouse anti-*β*-actin antibody as control. The membrane was washed five times for 5 min with PBS-Tween and incubated for 1 h at room temperature with the respective secondary antibodies conjugated to horseradish peroxidase (HRP). After five washes for 5 min with PBS-Tween buffer, the chemiluminescence of the labeled proteins was visualized with HRP substrate and captured using the LICOR Odyssey imaging system. The relative densities of proteins were all determined by using ImageJ (NIH) v.1.46r (Wayen Rasband, US National Institutes of Health, Bethesda, MD, USA).

### 2.7. Real-Time Quantitative PCR (RT-qPCR)

Total RNAs were extracted from MDCK cells at 6, 12, or 24 hpi, using the RNAfast200 kit (Fastagen Biotech, Shanghai, China) according to the Manufacturer’s instructions. Reverse transcription (RT) was carried out on 0.5 μg of total RNA by using PrimeScript RT reagent kit (Takara, Tokyo, Japan). Reverse transcription (RT) was conducted using specific oligonucleotides for vRNA (5′-AGC AAA AGC AGG-3′), cRNA (5′-AGT AGA AAC AAG G-3′), and mRNA [oligo(dT)]. A glyceraldehyde-3-phosphate dehydrogenase (GAPDH)-specific primer (5′-GAA GAT GGT GAT GGG ATT TC-3′) was also included in the RT reaction mixture for vRNA or cRNA analysis. The quantitative real-time PCR was performed in a 20-μL reaction mixture containing 50 nM forward and reverse primers (NP forward primer 5′-GAT TGG AAT TGG ACG AT-3′, reverse primer 5′-AGA GCA CCA TTC TCT CTA TT-3′; M1 forward primer 5′-AAG ACC AAT CCT GTC ACC TCT GA-3′, reverse primer 5′-CAA AGC GTC TAC GCT GCA GTC C-3′; M2 forward primer 5′- CCG AGG TCG AAA CGC CTA TC-3′, reverse primer 5′-CTT TGG CAC TCC TTC CGT AG-3′; NS1 forward primer 5′-CTT CGC CGA GAT CAG AAA TC-3′, reverse primer 5′-TGG ACC AGT CCC TTG ACA TT-3′; NS2 forward primer 5′-GTT GGC GAA ATT TCA CCA TTG CCT TCT CT-3′, reverse primer 5′-TTA AAT AAG CTG AAA TGA GAA AGT TCT-3′), 1 × SYBR green master mix (Takara Biotech, Dalian, China), and various amounts of template. To quantify the changes in gene expression, the change in threshold cycle (∆C_T_) method was used to calculate the relative changes normalized against the GAPDH gene (forward primer, 5′-AAT TCC ACG GCA CAG TCA AGG C-3′; reverse primer, 5′-AAC ATA CTC AGC ACC AGC ATC ACC-3′). The C_T_ was defined as the cycle at which fluorescence was determined to be significantly greater than the background. The ratio of viral RNA to the internal control was normalized to the control level 0 h after infection, which was arbitrarily set equal to 1.0.

### 2.8. In Vivo Experiments

Six to eight-week-old female BALB/c mice (average weight, 16.0 ± 2.0 g) were separated into 4 groups. Pretreatment was done by administering one dose of brevilin A (25 mg/kg or 10 mg/kg) or solvent (10% Lipovenos containing 0.2% DMSO, 10% PEG300 and 2.5% glycerol) intraperitoneally (i.p.) every other day in a volume of 0.1 mL for 6 days starting 1 hpi. The reference drug oseltamivir (20 mg/kg) was applied to mice orally via gavage once a day in a volume of 0.1 mL for 6 days starting 1 hpi. Mice were anesthetized with isoflurane (RWD R450, Shenzhen, China) and inoculated intranasally with 50 μL of a solution containing PR8 virus. Control animals were treated with distilled water. Body weight and survival were monitored daily for 14 days.

### 2.9. Statistical Analysis

Statistical significance was analyzed using GraphPad Prism 6 (GraphPad Software Inc.), and *, *p* ≤ 0.05; **, *p* ≤ 0.01; ***, *p* ≤ 0.001 were considered statistically significant. For paired samples, a paired *t* test was performed; otherwise, an unpaired Student *t* test was used. Differences in group survival were analyzed using Log-rank (Mantel-Cox) test. Error bars represent means ± standard deviations (SD).

## 3. Results

### 3.1. Brevilin A Shows a Broad-Spectrum Antiviral Activity against IAV

In our previous work, brevilin A showed potent antiviral activity against PR8 virus assessed by cytopathogenic effect (CPE) reduction assay and the cell viability assay [20]. To further verify its anti-IAV activity, brevilin A was tested in a plaque reduction assay using several IAV strains including A/PR/8/34 H1N1, A/FM/1/47 H1N1, A/Hong Kong/498/97 H3N2, and A/chicken/Guangdong/1996 H9N2 viruses. Ribavirin served as a positive control. The concentration for 50% of maximal effect (EC_50_) of brevilin A obtained with PR8 for viral plaque formation was calculated to be 2.96 ± 1.10 µM. This result concurs with the EC_50_ of 1.75 ± 0.59 µM that we evaluated in previous work. Comparable to PR8, the EC_50_ values of brevilin A obtained with H1N1 (FM1), H3N2, and H9N2 were 1.60 ± 1.14, 3.28 ± 1.09, and 2.07 ± 1.12 µM, respectively (Table 1). While the EC_50_ of ribavirin obtained with these four IAV strains were between 7.05 to 10.76 µM. These results indicate that brevilin A exhibits better anti-IAV activity than ribavirin, and the effects of both are not IAV type/subtype specific. In order to test whether brevilin A possesses antiviral activity against other RNA viruses, the effect of brevilin A on respiratory syncytial virus (RSV) was evaluated by a CPE reduction assay. However, brevilin A did not show inhibitory effect on RSV at a noncytotoxic concentration.

### 3.2. Brevilin A Inhibits Progeny Virus Production in Various Virus-To-Cell Ratios

**Figure 2:**
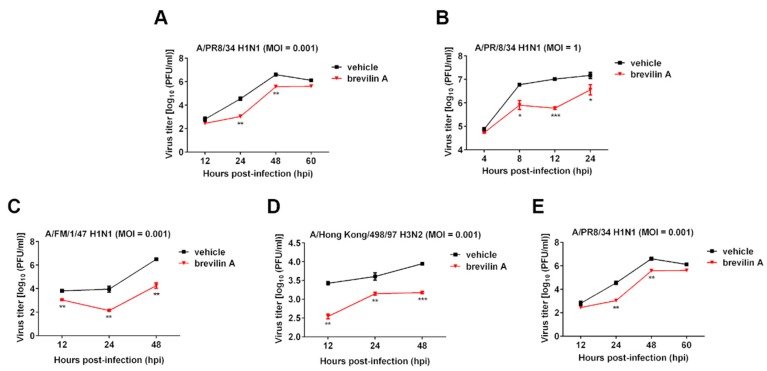
The inhibitory effect of brevilin A on the growth curves of various influenza A viruses (IAV) strains. Madin–Darby canine kidney (MDCK cells) were infected with influenza A/PR/8/34 H1N1 virus at a MOI of 0.001 (**A**) or 1 (**B**), or A/FM/1/47 H1N1 virus (**C**), A/Hong Kong/498/97 H3N2 virus (**D**), or A/chicken/Guangdong/1996 H9N2 virus (**E**) at a MOI of 0.001. Cells were then treated with 8 µM of brevilin A or vehicle. At the indicated time points after infection, virus titers in the supernatants were determined by a plaque assay. The data represent means ± SD. *, *p* < 0.05; **, *p* < 0.01; ***, *p* < 0.001 are considered statistically significant, compared to vehicle.

To examine to what extent the anti-IAV activities of brevilin A is affected by virus-to-cell ratio, the cells were infected with PR8 at a MOI (MOI, defined as the ratio of input infectious viral particles per target cell) of 0.001 or 1 in the presence of either brevilin A (8 µM) or vehicle control (DMSO). Virus titers in the supernatants at the indicated time points were quantified by plaque assays. As shown in Figure 2A, after infection with virus at a MOI of 0.001, the amount of progeny virus in the supernatants increased over the incubation time and peaked at 48 hpi in vehicle control, while treatment with brevilin A could significantly reduce the production of infectious virus from cells at 24 or 48 hpi. Even when cells were infected with virus at a higher MOI (MOI = 1), treatment of brevilin A also significantly decreased virus production by about 10-fold at 8 and 12 hpi (Figure 2B). These findings imply that the treatment of brevilin A strongly suppresses the replication of IAV, of note, the inhibitory activity of brevilin A is still rather effective against a relatively higher dose of input virus.

Additionally, we also analyzed the impact of brevilin A on replication of H1N1 (FM1), H3N2, or H9N2 in MDCK cells over multiple replication cycles. As shown in Figure 2C–E, compared to the vehicle, virus titers at each time point were markedly reduced by treatment with brevilin A.

### 3.3. Brevilin A Is Effective at the Viral Genome Replication and Translation Stage

**Figure 3:**
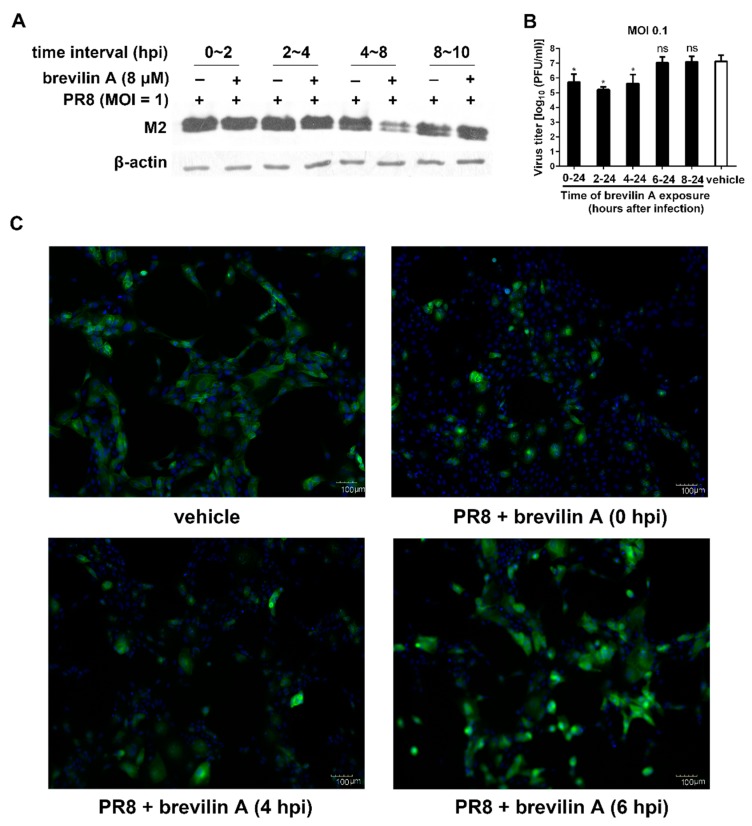
Influence of different treatment conditions of brevilin A on IAV replication. (**A**) MDCK cells were infected with PR8 at a MOI of 1, brevilin A or vehicle was present at four time-intervals, 0–2, 2–4, 4–8, and 8–10 hpi. At 12 hpi, cell lysates were analyzed by Western blot assay; (**B**) MDCK cells were infected with PR8 at a MOI of 0.1, then treated with brevilin A (8 µM) at 0, 2, 4, or 6 hpi. The virus titers in the supernatant were determined by plaque assay at 24 hpi; (**C**) MDCK cells infected with PR8 (MOI = 1) were treated with brevilin A at the indicated times. The M2 protein expression was determined by immunofluorescence assay at 12 hpi. The scale bar in the images is 100 μm. The data represent means ± SD. *, *p* < 0.05 is considered statistically significant compared to vehicle.

The life cycle of influenza virus is around 8–10 h and is divided into three steps: virus entry (0–2 h), viral genome replication and translation (2–8 h), and progeny virion release (8–10 h) [23]. To investigate the stage of viral cycle where brevilin A exhibits its activity against PR8 virus, we next performed time of addition experiments. MDCK cells were infected with PR8 virus, and brevilin A was added or removed at the indicated time points. The expression levels of influenza M2 protein in infected cells were measured at four time-intervals, 0–2, 2–4, 4–8 and 8–10 hpi. We found that the M2 level at the interval 4–8 hpi was reduced around 70%, compared to the vehicle. In contrast, no antiviral activity was detected for the remaining three intervals (0–2, 2–4, 8–10 hpi) (Figure 3A). These data indicate that brevilin A is effective at the viral genome replication and translation stage. No inhibitory effect was observed at virus entry stage or progeny virion assembly/release stage.

We then performed two other experiments to examine the mode of action of brevilin A. First, brevilin A was added to the IAV infected cells at 0, 4, 6, or 8 hpi, the supernatant was collected at 24 hpi, and the virus titers were determined by plaque assay. The virus titers were decreased when brevilin A was added at 0–4 hpi, compared to vehicle. However, similar titers were observed in the treatment and vehicle control when the compound was added 6 h after infection (Figure 3B). Moreover, the results obtained with an immunofluorescent assay showed that compared to the vehicle control, the expression of viral protein M2 was markedly reduced when brevilin A was added at 0 to 4 hpi, and addition of brevilin A at 6 hpi still had a minor impact on M2 expression (Figure 3C). These data suggest that brevilin A blocks the intermediate stage (s) of the influenza virus life cycle between approximately 4 to 6 hpi.

### 3.4. Brevilin A Inhibits Influenza Viral RNA Synthesis

**Figure 4:**
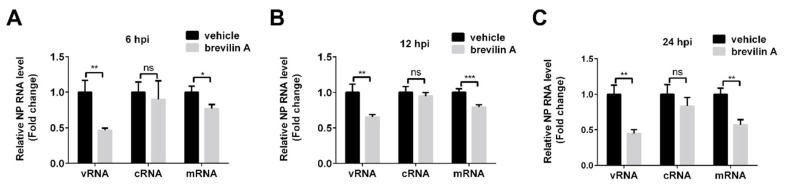
The inhibitory effects of brevilin A on the expression of virus RNA. MDCK cells were infected with PR8 at a MOI of 1, then treated with brevilin A or vehicle. Total RNA was extracted at 6 hpi (**A**), 12 hpi (**B**), or 24 hpi (**C**), and the vRNA, cRNA, and mRNA were quantified by RT-qPCR. *, *p* < 0.05; **, *p* < 0.01; ***, and *p* < 0.001 are considered statistically significant, compared to vehicle.

Transcription of vRNA produces mRNA and complementary RNA (cRNA). The former serve as the template for synthesis of viral proteins, and the latter are templates for synthesis of more vRNA for production of new virions [24]. We next performed RT-qPCR to evaluate the effect of brevilin A on viral RNA synthesis. MDCK cells were infected with PR8 at 1 MOI, then treated with brevilin A or DMSO. Total RNA was extracted at 6, 12, or 24 hpi and reverse transcription was performed with specific primers for viral NP gene. At 6 hpi, when the viral RNA synthesis was already completed [25], the levels of vRNA in brevilin A-treated samples were significantly reduced in comparison to those in vehicle treated cells by 50%. In contrast, the levels of mRNA were only decreased by ~20% and cRNA was not altered upon the treatment with brevilin A (Figure 4A). We next tested several later time points post-infection to determine the inhibitory effects of brevilin A during multiple replication cycles of IAV. The effect of brevilin A on cRNA levels was not significant at 12 or 24 hpi (Figure 4B). However, the inhibition of mRNA levels became notable at 24 hpi with a reduction of about 45% (Figure 4C). These results indicate that the inhibitory effect of brevilin A is intimately involved with the vRNA synthesis.

### 3.5. Brevilin A Decreases the Levels of Viral mRNA and Proteins Expressed from the M and NS Segments

**Figure 5:**
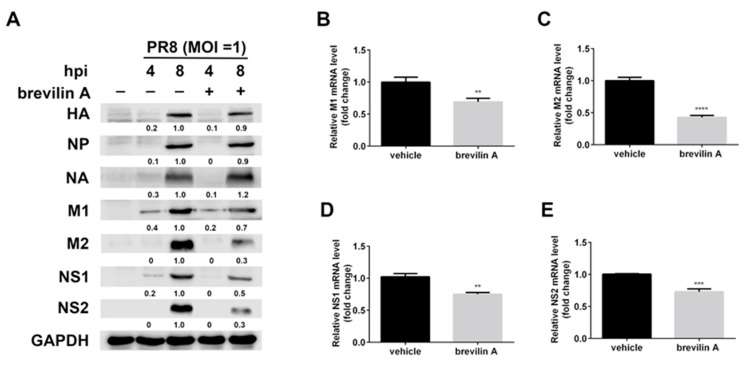
The inhibitory effects of brevilin A on the mRNA and protein expression of viral NS and M. (**A**) MDCK cells were infected with PR8 at a MOI of 1, and then treated with brevilin A or vehicle. At the indicated time points, cell lysates were analyzed by Western blot assay for the indicated antigens; (**B**–**E**) MDCK cells were infected with PR8 at a MOI of 1, and then treated with brevilin A (8 µM) at 1 hpi, total RNA was extracted from the infected cells at 6 hpi, and mRNA was quantified with RT-qPCR. **, *p* < 0.01; ***, *p* < 0.001; ****, and *p* < 0.0001 are considered statistically significant, compared with vehicle.

Next, we investigated the expression pattern of viral proteins during IAV infection observed in the presence of brevilin A. For this purpose, MDCK cells were infected with PR8 (MOI = 1) and treated with brevilin A at a concentration of 8 µM. At the time point of 4 hpi, only the expressions of NA, M1, and NS1 were observed in the control cells. Treatment with brevilin A resulted in about 20% reduction of these proteins (Figure 5A). At 8 hpi, the expressions of viral NA, HA, and NP were not affected by brevilin A, and a modest reduction of M1 (~30%) was observed upon brevilin A treatment. However, the reduction of M2, NS1, and NS2 proteins was much more dramatic: about 70% in M2, 50% in NS1, and 70% in NS2, respectively (Figure 5A). The M2 protein is translated from M2 mRNA, which is produced from the alternative splicing of M1; the NS1 and NS2 proteins were respectively translated from NS1 and NS2 mRNA generated from the NS segment. We next performed RT-qPCR to determine the effect of brevilin A on the mRNA expressions of M1, M2, NS1, and NS2. We infected MDCK cells with PR8 virus and then treated cells with brevilin A. Whole-cell RNA was isolated from the infected cells at 6 hpi and quantified for mRNA using RT-qPCR assay. The M2 mRNA was markedly reduced following treatment with brevilin A, with a reduction of 55%–60%, whereas the reduction rates of M1, NS1, and NS2 mRNA levels were similar to that of NP (with a reduction of ~20%) compared to vehicle (Figure 5B–E). These results suggest that brevilin A reduces the mRNA and protein expressions of viral M and NS.

### 3.6. Brevilin A Induces Influenza Viral RNP Aggregation in the Nucleus

**Figure 6:**
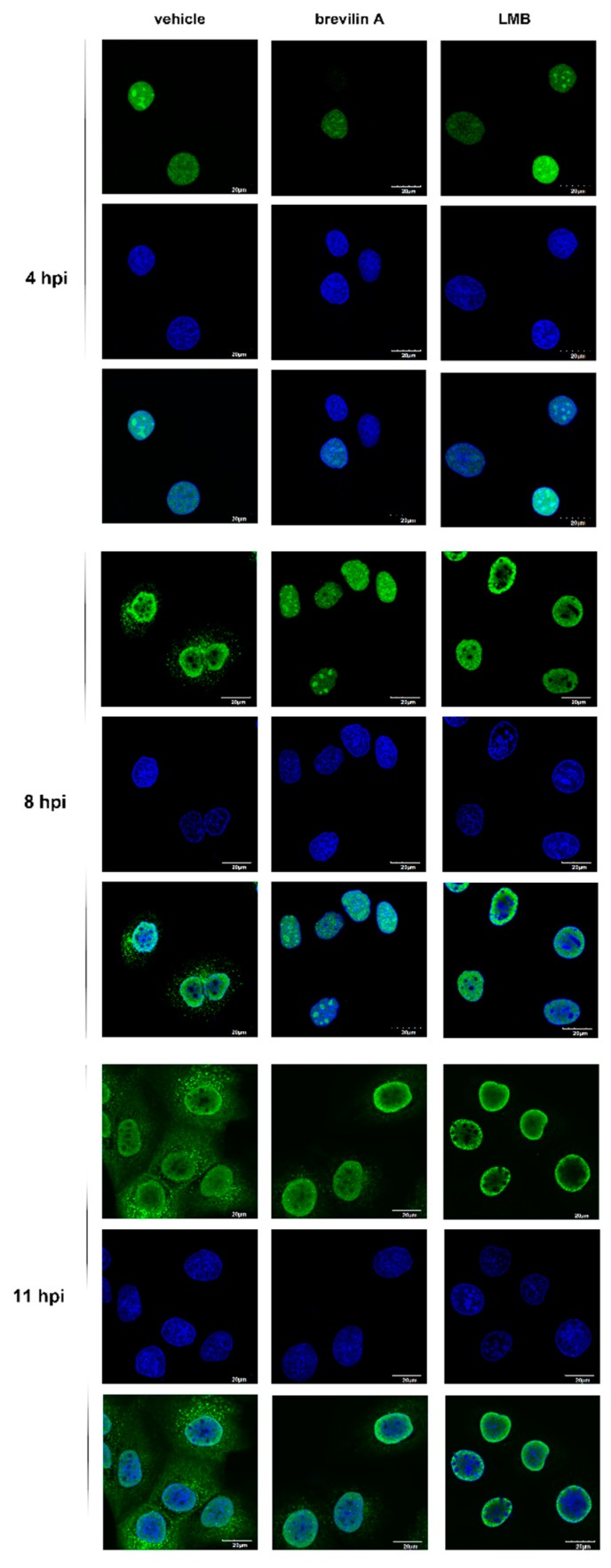
The effects of brevilin A on the viral ribonucleoproteins (vRNP) localization. MDCK cells were infected with PR8 at a MOI of 3 and then treated with brevilin A, LMB, or vehicle as indicated. Samples were fixed at 4, 8, or 11 hpi, and then stained with anti-NP body (green) and DAPI (blue). Immunofluorescence was observed with confocal microscopy. The scale bar in the images is 20 μm.

In contrast with most RNA viruses, the replication and transcription of IAV were carried out in the nucleus of the infected cells. After uncoating, the vRNP complex, which consists of the viral PB1-PB2-PA (3P) heterotrimeric RNA polymerase and NP protein, is imported into the nucleus for virus RNA replication and transcription and then exported to the cytoplasm for packaging into newly formed virions at the cellular plasma membranes [26]. The matrix protein M1, the viral nuclear export protein (NS2/NEP), and the M2 ion channel protein are essential proteins involved in viral trafficking, releasing into the cytoplasm, and budding [27]. To investigate the effects of brevilin A on the nucleocytoplasmic trafficking of vRNPs, MDCK cells were infected with PR8 virus for 1 h and then treated with DMSO, brevilin A, or LMB. The viral NP protein was detected by indirect immunofluorescence microscopy at 4, 8, and 11 hpi to determine the vRNP localization. As shown in Figure 6, in vehicle treated cells, the NP protein was detected exclusively in the nucleus at 4 hpi and shifted toward the cytoplasm at 8 hpi. By 11 hpi, vRNPs were mainly distributed in the cytoplasm. As a control, we used LMB, a potent and specific nuclear export inhibitor, which has been demonstrated to be able to cause the nuclear accumulation of newly generated vRNPs [28]. Consistent with the literature report, LMB prevented the export of vRNPs, even at the late stage of infection (11 hpi), whereas their nuclear import was not affected. Similar to LMB, upon treatment with brevilin A, the translocation of vRNPs to the nucleus was delayed. The aggregation of vRNPs was also observed at 11 hpi (Figure 6). These results suggest that brevilin A induces vRNPs aggregation in the nucleus.

### 3.7. Brevilin A Protects Mice from IAV Pathogenesis

**Figure 7:**
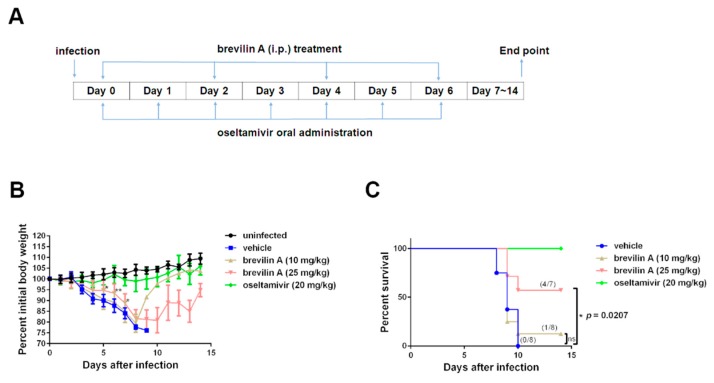
Brevilin A decreases the lethality observed in IAV-infected mice. (**A**) BALB/c mice were infected intranasally with three 50% lethal doses (LD_50_) of PR8 virus. Brevilin A (25 mg/kg or 10 mg/kg) was intraperitoneally (i.p.) administered to mice 1 h after virus infection, and then once every other day for 6 days beginning on the day of infection (*n* = 8). Oseltamivir phosphate (20 mg/kg) used as a positive control was administered by oral gavage every day (*n* = 7). Vehicle (10% Lipovenos containing 0.2% DMSO, 10% PEG300 and 2.5% glycerol, *n* = 8) was used as a negative control. (**B**) The body weight of mice from each group was monitored daily from day 0 to day 14. The data represent means ± SD. (**C**) The survival rates of the mice were calculated. Animals falling below the threshold of 75% of their initial body weight were humanely euthanized. The *p*-value is shown (Log-rank (Mantel–Cox) test).

Since brevilin A showed antiviral activity against influenza A virus in vitro, we next examined whether brevilin A could also protect mice against influenza virus infection. Mice were infected intratracheally with PR8 at a dose of three 50% lethal doses (LD_50_). Brevilin A was given once every other day intraperitoneally immediately after infection at two concentrations (10 mg/kg or 25 mg/kg). Oseltamivir was used as the positive control that it is commonly used for treating influenza virus infection in the clinic. The experiment was conducted following the scheme illustrated in Figure 7A. Morbidity and mortality were monitored daily by measuring the body weight and survival rate (Figure 7B,C). Animals falling below the threshold of 75% of their initial body weight were humanely euthanized. Vehicle-treated mice showed severe morbidity after infection with influenza virus, and 100% mortality at 10 days post-infection, while the uninfected group (normal) and oseltamivir-treated group (20 mg/kg/day) showed 100% survival during the entire experiment. Mice treated with brevilin A at 10 mg/kg did not show significant differences in terms of body weight loss or survival rate (Figure 7B,C). However, treatment of brevilin A at 25 mg/kg sustained the body weights of mice in comparison to vehicle-treated group (Figure 7B). Also, brevilin A-treated mice (25 mg/kg) showed a delayed time-to-death with 50% survival up to 14 days post-infection (Figure 7C). Thus, these results show that brevilin A protects mice from IAV pathogenesis.

## 4. Discussion

The present study shows that brevilin A at a noncytotoxic concentration has a broad-spectrum antiviral activity against IAV, including H1N1, H3N2, and H9N2. Mode of mechanism studies demonstrate that brevilin A exhibits its antiviral activity by regulating the replication and translation stages of IAV life cycle. Brevilin A strongly decreased viral RNA level, reduced the expression of viral proteins expressed from the smaller segments (M and NS), and impaired the nuclear export of vRNPs. Furthermore, we showed that brevilin A reduced influenza-associated morbidity and mortality in vivo.

In the current study, we determined that the anti-IAV activity of brevilin A is not viral subtype specific as brevilin A displayed a broad-spectrum antiviral activity against many IAV types/subtypes. These effects were assessed by plaque reduction assay and generation of virus growth curves (Figure 2). Using a time-of-addition assay, we deduced that brevilin A acts at the replication and translation stages of infection (Figure 3), which could explain why the virus titers at 4 hpi were not reduced by treatment with brevilin A (Figure 2B). Further examination reveals that brevilin A preferentially regulates the synthesis of vRNA but not the complementary positive strand cRNA or the mRNA (Figure 4). Moreover, by analyzing the expression of viral protein, we determined that not all of the viral proteins are equally affected by brevilin A. Expression of M2, NS1, and NS2 proteins are more severely inhibited in comparison to other viral proteins (Figure 5A). It has been reported that some IAV genes (segments 1, 2, 3, 5, and unspliced 8) are preferentially expressed early and the others (segments 4, 6, unspliced 7, and two spliced transcripts) are expressed late during infection [23]. However, this could not explain our observation that at early time points post-infection, the proteins translated from the M and NS mRNAs were down-regulated by treatment with brevilin A, while the expression of HA and NA (supposed to be preferentially expressed early) were not affected. Considering that the M and NS segments are coincidentally the ones that produce spliced products, the mRNA levels of M1, M2, NS1, and NS2 were analyzed. Our results demonstrate that the production of M2 mRNA was strongly reduced following the treatment with brevilin A (Figure 5B–E), indicating that the alternative splicing of the M1 mRNA was affected. Splicing is a necessary step for influenza replication, while NS2 is required for nucleocytoplasmic transport of vRNPs [29], and M2 is an important factor in viral pathogenicity [30,31]. During the nuclear replication stage, numerous host-splicing factors, such as the spliceosome complex and host splicing regulators, are necessary to process the M and NS segments [32]. How brevilin A affects the alternative splicing of influenza A viruses needs to be further investigated.

Brevilin A’s antiviral effects in vivo were also evaluated in a mouse model upon influenza virus infection. Often, a compound possessing potent inhibitory activity in vitro fails when tested in vivo. However, we found that treatment of IAV-infected mice with brevilin A markedly improved their survival, compared to vehicle control mice (Figure 7). Under our experimental settings, brevilin A (25 mg/kg) was delivered every other day for four times rather than every day for 6 days, since both delivery frequency showed similar protective effects in IAV-infected mice. Further exploration of the in vivo potential of brevilin A is warranted to assess its in vivo toxicity, and define the best conditions of treatment, including analysis of dosage and route(s) of inoculation. Besides, brevilin A’s bioavailability and pharmacokinetics would also be considered in future research to get more detailed information about absorption, metabolism, and disposition.

In summary, the sesquiterpene lactone brevilin A is a promising candidate lead compound for development of antiviral agents that broadly inhibit IAV replication by impairing the vRNA synthesis and the viral protein translation. Further investigation is warranted of this and other similar inhibitors as potential therapeutic agents against influenza.

## Figures and Tables

**Table 1:** Anti-IAV activities of brevilin A.

Comp.	IAV	EC_50_ (µM) *^a^*	SI *^b^*
**brevilin A**	A/PR/8/34 H1N1	2.96 ± 1.10	8
	A/FM/1/47 H1N1	1.60 ± 1.14	14
	A/Hong Kong/498/97 H3N2	3.28 ± 1.09	7
	A/chicken/Guangdong/1996 H9N2	2.07 ± 1.12	11
**ribavirin**	A/PR/8/34 H1N1	7.05 ± 1.10	>14
	A/FM/1/47 H1N1	9.19 ± 1.02	>20
	A/Hong Kong/498/97 H3N2	10.76 ± 1.07	>18
	A/chicken/Guangdong/1996 H9N2	10.35 ± 1.04	>18

*^a^* Effective concentration required for reducing virus-induced plaque number by 50%. *^b^* Selectivity index, CC_50_/EC_50_.
